# Supplementary material for: Identifying topology of leaky photonic lattices with machine learning
Source: Nanophotonics. 2024 Jan 24;13(3):271–81. doi: 10.1515/nanoph-2023-0564 (PMC11501594; doi:10.1515/nanoph-2023-0564)
Supplement: Supplementary file 1 — Supplementary Material Details [file j_nanoph-2023-0564_suppl_001.pdf]

Supplementary Materials for

"Identifying topology of leaky photonic lattices with machine learning"

I. ARRAYS AND PARAMETERS

In this section, we first derive dispersion relations [Eqs. (5) in the main text] for the main (SSH) and the environmental arrays.

The infinite SSH model, non-interacting with the environmental array, is described by the system of coupled equations:

$$i\frac{\partial \psi_m}{\partial z} = J_1\psi_{m-1} + J_2\psi_{m+1}, \quad m-\text{even}, \tag{S1a}$$

$$i\frac{\partial \psi_m}{\partial z} = J_2\psi_{m-1} + J_1\psi_{m+1}, \quad m-\text{odd}\,. \tag{S1b}$$

The unit cell of the SSH lattice consists of two elements and, therefore, we can rewrite the system in terms of the two-component wavefunction $[\psi_n^{(1)}, \psi_n^{(2)}]$, where n is the number of the unit cell:

$$i\frac{\partial \psi_n^{(1)}}{\partial z} = J_1\psi_n^{(2)} + J_2\psi_{n-1}^{(2)}, \tag{S2a}$$

$$i\frac{\partial \psi_n^{(2)}}{\partial z} = J_1\psi_n^{(1)} + J_2\psi_{n+1}^{(1)}. \tag{S2b}$$

Applying the Fourier transform $[\psi_n^{(1)}, \psi_n^{(2)}] \sim [\Psi_1, \Psi_2]\exp(in\kappa_y L_y)$, where L_y is a spatial period and κ_y is the wavenumber, to Eqs. (S2), we obtain the Bloch Hamiltonian in the momentum representation:

$$\hat{H}^{\text{SSH}}(k) = \begin{pmatrix} 0 & J_2 e^{-i\kappa_y L_y} + J_1 \\ J_2 e^{i\kappa_y L_y} + J_1 & 0 \end{pmatrix}. \tag{S3}$$

Its eigenvalues,

$$\beta_{\text{SSH}}^{(\pm)} = \pm\sqrt{J_1^2 + J_2^2 + 2J_1J_2\cos\kappa_y L_y} \equiv \pm\sqrt{J_1^2 + J_2^2 + 2J_1J_2\cos\varkappa},$$

determine the propagation constants of the modes in the SSH waveguide array. Here, we denote the dimensionless wavenumber $\varkappa = \kappa_y L_y$.

Next, the coupled equations for the environmental array are

$$i\frac{\partial c_l}{\partial z} = \Delta c_l + J_{\text{env}}(c_{l-1} + c_{l+1}). \tag{S4}$$

Applying the Fourier transform $c_l \sim C\exp(il\kappa_x L_x)$, where L_x is a spatial period, we obtain the single-band dispersion relation

$$\beta_{\text{env}} = \Delta + 2J_{\text{env}}\cos\kappa_x L_x \equiv \Delta + 2J_{\text{env}}\cos\varkappa,$$

where $\varkappa = \kappa_x L_x$.

By fitting the band diagrams calculated in the framework of the paraxial equations, we can obtain the parameters of the model Hamiltonians, the coupling coefficients $J_{1,2,\text{env}}$ and detuning Δ. The ranges of variation in the parameters are summarized in Table 2 in the main text. The full intersection of the environmental array's dispersion curve with the main lattice's lower band is ensured by these values. The corresponding condition can be formalized as follows:

$$\left(\max\beta_{\text{env}}(\varkappa) < \min\beta_{\text{SSH}}^{(+)}(\varkappa)\right) \cap \left(\max\beta_{\text{env}}(\varkappa) > \max\beta_{\text{SSH}}^{(-)}(\varkappa)\right) \cap \left(\min\beta_{\text{env}}(\varkappa) < \min\beta_{\text{SSH}}^{(-)}(\varkappa)\right), \tag{S5}$$

which is equivalent to

$$(\Delta + 2J_{\text{env}} < |J_1 - J_2|) \cap (\Delta + 2J_{\text{env}} > -|J_1 - J_2|) \cap (\Delta - 2J_{\text{env}} < -|J_1 + J_2|) \tag{S6}$$

or

$$(-|J_1 - J_2| < \Delta + 2J_{\rm env} < |J_1 - J_2|) \cap (\Delta - 2J_{\rm env} < -|J_1 + J_2|)\,. \tag{S7}$$

For comparison of topological and non-topological cases, we also introduced the two-band non-topological lattice with dispersion

$$\beta_{\text{non-top}} = 1/2(\Delta_1 + \Delta_2 \pm \sqrt{(\Delta_1 - \Delta_2)^2 + 8J^2 + 8J^2 \cos \varkappa})$$

and parameters changing in the ranges $\Delta_1 \in [0.9, 1.2]$, $\Delta_2 = -\Delta_1$, $J \in [0.7, 1.2]$. In Fig. S1, we visualize the corresponding variations in the propagation constants. As deliberately ensured, the environmental array's dispersion curve fully intersects the lower band of the two-band lattice only. Such an intersection of the bands of the main and auxiliary lattices indicates auxiliary arrays act as leaky channels leading to the loss of energy in the lower band. Therefore, if a wavepacket short in real space (or equivalently, long in the reciprocal space, composed of almost all the wave vectors) is excited initially, only the modes of the upper band will be retained in a finite propagation distance.

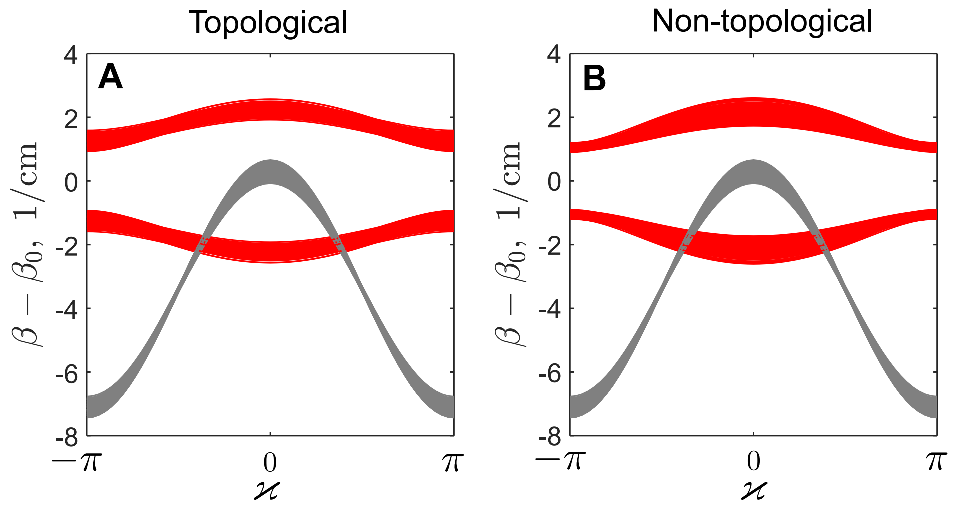

FIG. S1. Propagation constant β for the topological (A) and non-topological (B) lattices within the variation limits. The bands of the main array are plotted in red color, the dispersion curve of the environmental array – in gray color.

The parameters of the TBM model were chosen to match the band diagrams and dynamics acquired through the paraxial modeling (see Fig. S2). Slight distinctions between these two models are related to the effect of the next-nearest-neighbor interactions. However, these distinctions appear insignificant for our problem formulation.

Next, we discuss the relationship between the TBM coupling strength and corresponding parameters of the realistic laser-written array. Our assumption is that the strength of the coupling varies depending on the distance between neighboring waveguides (distance d). We then model a dimer of waveguides and estimate the coupling strength J as a half-difference between the propagation constants of the symmetric and antisymmetric eigenmodes. We observe the expected exponential decay of J with increasing d (see the fitting dashed black exponential curve in Fig. S3).

To demonstrate the effects of the chiral disorder, which involves the addition of random variables to the coupling coefficients, we perform the following steps. In the main text Section 5, we introduce off-diagonal disorder by incorporating a random variable to the coupling coefficients, where the value is proportional to $l\langle d\rangle \text{mean}(J_1, J_2)$. Here, l follows a uniform distribution in the range $[-1/2, 1/2]$ and $\langle d\rangle$ represents the disorder strength. First, we fix the coupling coefficients to be $J_1 = 1.75$ cm^{-1} and $J_2 = 0.5$ cm^{-1}, and then add the maximum amplitude of the random disorder, as $J_{1,2} \pm 0.1(1.75 + 0.5)/2 \times 0.5$, where $\langle d\rangle = 0.1$, $\text{mean}(J_1, J_2) = (1.75 + 0.5)/2$, $l = 0.5$. This maximum value of the disorder strength, $\langle d\rangle = 0.1$, corresponds to a shift of the center of the inter-waveguide distance to a value $\approx 0.5\ \mu m$.

II. DISCUSSION OF THE T-SNE METHOD RESULTS

In this section, we analyze the statistics of the intensity distributions to provide further insights into the details of the t-SNE method. Figure S4 illustrates the normalized histograms, representing the statistical characteristics of

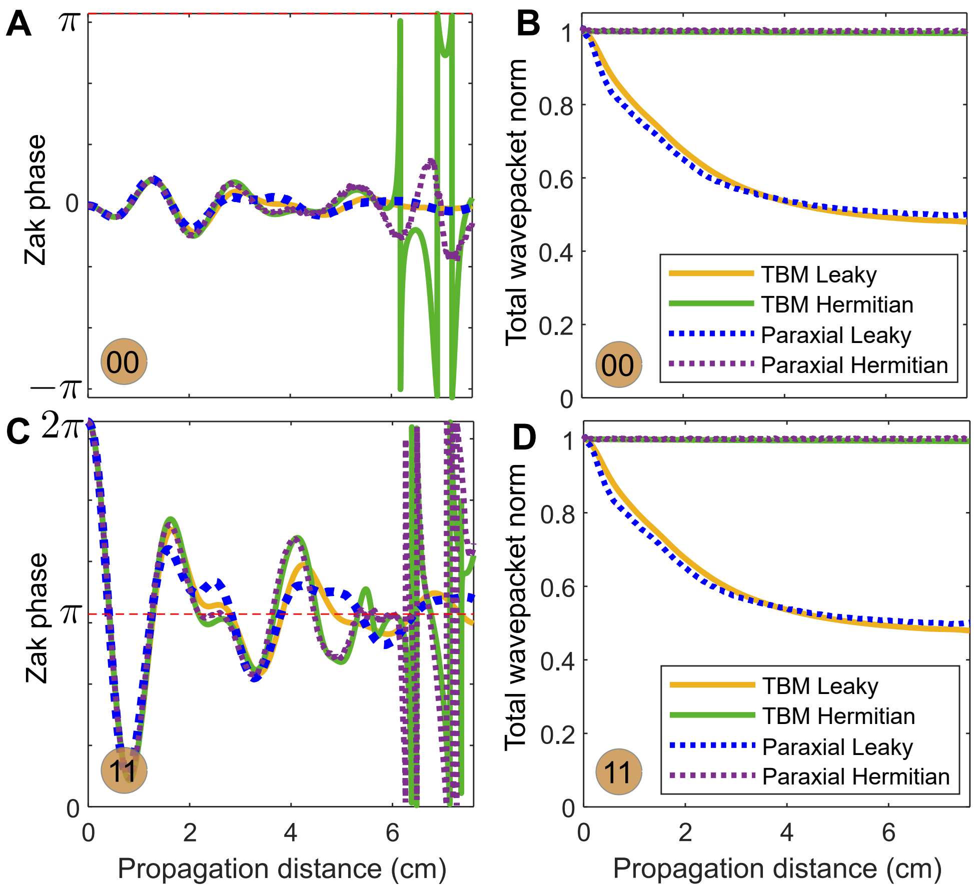

FIG. S2. Example of evolution dynamics in the paraxial model and tight-binding approximation for the Hermitian SSH lattice and SSH lattice with leaky channels.

the intensity distribution from $N = 22(23)$ waveguides for the range of TBM coupling strengths as in Table 2. These distributions were formed by propagating the field to the fixed distance $L = 7.6$ cm. The resulted data serves as input for unsupervised learning with t-SNE, where the vector's dimension depends on the respective topology and ranges from 22 to 23.

Using Fig. S4, our goal is to understand how the t-SNE method distinguishes between different topological classes. First, it should be noted that classes with identical edge topologies (00 and 11) are distinguishable from 01 and 10 due to varying input vector lengths (22 and 23 respectively). Furthermore, the distinction between classes 01 00 and 11 10 can be made by analyzing the maximum values of their respective intensity distributions, which are visibly different. In the Hermitian case, these statistical distributions do not have the pronounced maxima, making the topological class identification less straightforward, which is also noticeable in Fig. 3 in the main text. By contrast to the non-Hermitian case, the boundaries between areas representing different topological classes are less distinct in the Hermitian lattices. Consequently, it becomes challenging to accurately determine the topological class when the initially excited waveguide can vary, as shown in Fig. 3 (C).

Using this approach utilizing statistics, we can also explain the appearance of 6 classes in Fig. 3(F) in the main text for the leaky lattice. We offer additional elaboration on the method previously mentioned in the main text, which involves labeling not just the four topological classes but also marking the initially excited waveguide. As we can see, the method does not mark the difference between different initial excitations for topologies 00 and 01, as depicted in Fig. S5, which is in accordance with Fig. S4, where maxima of the distributions for different i are similar in these cases (see blue and black curves in the first and second columns).

While the introduction of leakage channels in the photonic lattice leads to more distinguishable scenarios compared to the Hermitian case, it is still not possible to identify the topology solely based on the distributions of the central elements. Consequently, the utilization of supervised machine learning methods becomes necessary.

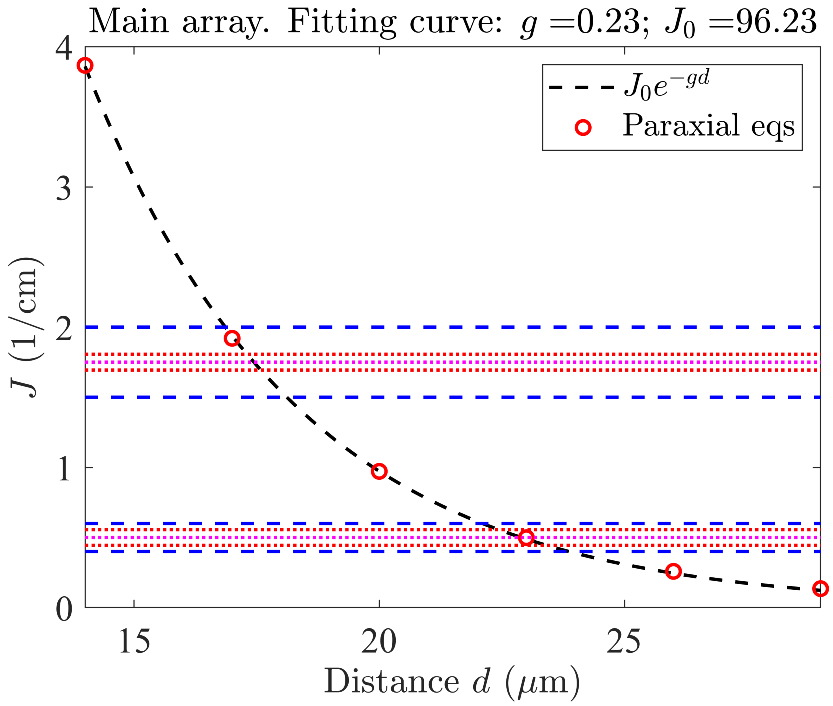

FIG. S3. Coupling strength as a function of the distance between centres of waveguides $J(d)$ calculated using the paraxial equations (red dots). The horizontal blue dashed lines mark the ranges $J_2 \in [1.5, 2]$ and $J_1 \in [0.4, 0, 6]$ (see Table 3 in the main text). The magenta lines are $J_1 = 0.5$ and $J_2 = 1.75$, and red dotted lines depict the ranges of disordered coupling coefficients for $\langle d \rangle = 0.1$.

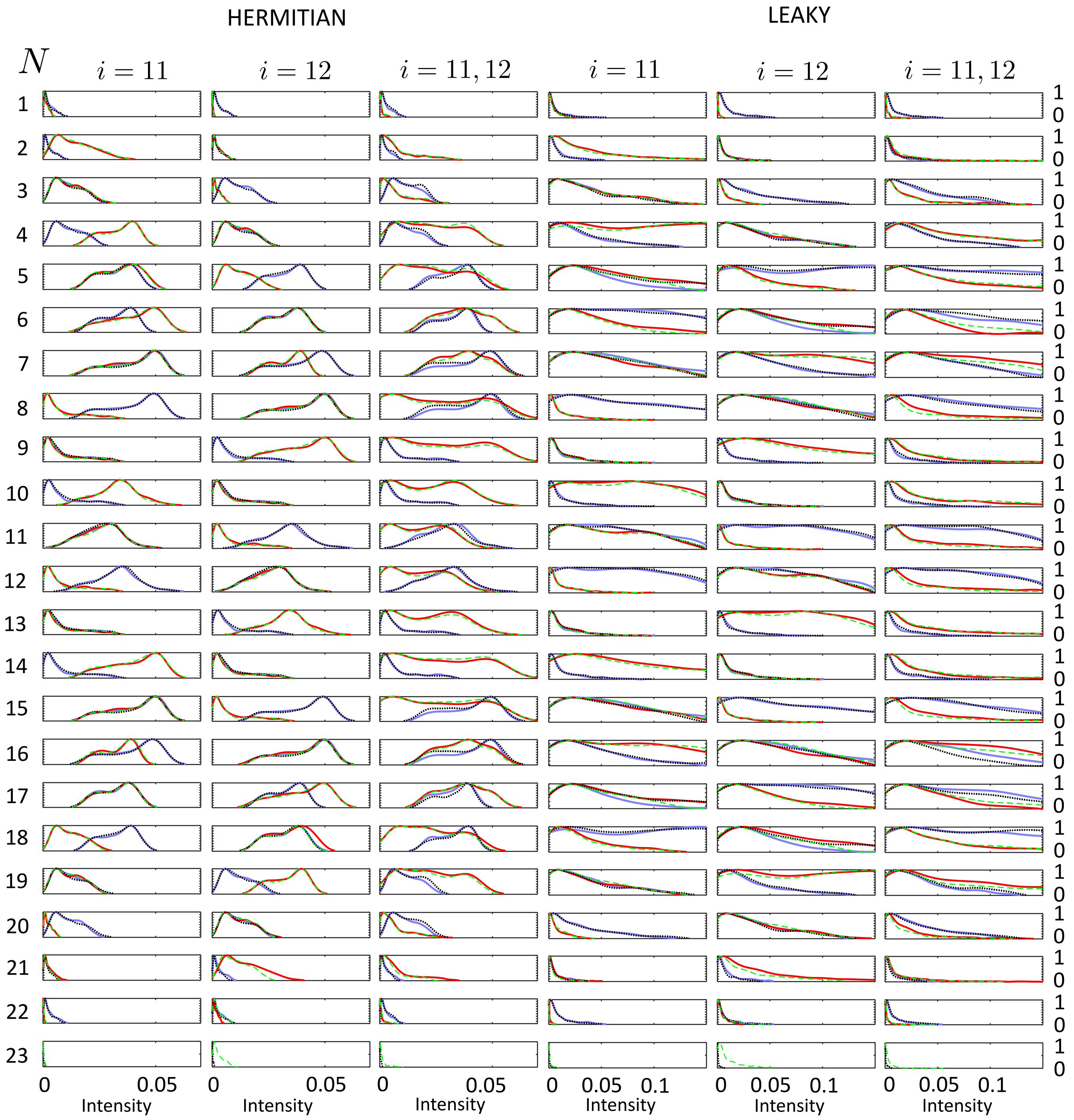

FIG. S4. Statistical characteristics of intensity distributions for different waveguides ($N = 1, ..., 23$) in different topological classes (explanation of Fig.3 in the main text). Columns 1-3 depict the case of the leaky lattice, while columns 4-5 represent the Hermitian one. To denote the number of initially excited waveguides, we mark it with the index i. Specifically, columns 1-2 and 4-5 correspond to the excitation of a single waveguide, and the datasets used to plot columns 3 and 6 were prepared for the two possible initial excitations. The topological class 00 is represented by the blue color, while the red color represents class 11. Class 01 is depicted by the black color, and the green color corresponds to class 10 (as in Fig. 3 in the main text).

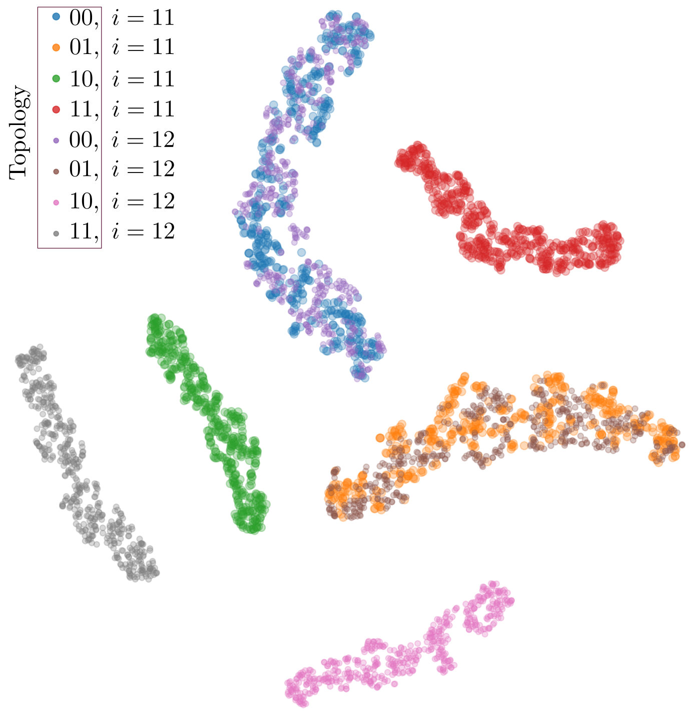

FIG. S5. t-SNE maps of the system with leaky channels having 4 topological classes depending on its 2 edge (as in Fig. 3(E)), then the excited waveguide is randomly chosen within a dimer (number of the excited waveguide is $i = 11$ or 12). In the legend, we write the type of topology (00,11,01,10) and the number of the initially excited waveguide i.

III. DESCRIPTION OF MACHINE AND DEEP LEARNING METHODS

- **K-Nearest Neighbors** (KNN)

  This classical machine learning algorithm is based on finding the nearest neighbors from the train dataset for each point (sample) of the classified set (query/test dataset). Hereby, data on the classes of the nearest neighbors is used to predict the class of the point (this can be done in different ways). [S1]

- **Decision Tree**

  The tree-like algorithm of classical machine learning is based on the spatial separation of samples of the training data set according to individual features. Going down the tree, each node of the algorithm chooses a feature by which the data will be divided and beats the set according to the selected criteria. [S2]

- **Support Vector Machine** (SVM)

  Binary classification algorithm of classical machine learning based on iterative selection of a separating plane in some space. This space is determined by setting the kernel function (kernel function), which sets the distance metric between two individual points. The separating plane itself is set by the so-called support vectors and must satisfy the max margin criterion. Thus, the binary class for the point $\mathbf{x} \in \mathbb{R}^n$ under study is determined by the sign of the linear combination λ_i of the set of answers y_i of the support vectors $\mathbf{x}_i$ and the distance (taking into account the function kernels) to them $K(\mathbf{x}, \mathbf{x}_i)$.

  $$y(\mathbf{x}) = \mathrm{sign}\left(\sum_{i=1}^{s} \lambda_i y_i K\left(\mathbf{x}, \mathbf{x}_i\right) - w_0\right).$$

  Note that this is the full equivalent of a fully connected neural network having n input neurons and s neurons in the hidden layer. In this way one can explain the similar accuracy trends for MLP & SVM in the graphs.

To achieve the best quality on the validation set, we performed selection of hyperparameters for all machine learning methods. The tables below contain the best results obtained during the selection.

Parameter for KNN	Range
n_neighbors	$[3, 5, 7, 9]$
weights	[uniform, distance]

TABLE I. Hyperparameters for KNN.

Parameter for SVM	Range
kernel	[linear, rbf, poly]
C	$[1, 2, 3, ..., 10, 15, 20, 25, 30, 100, 200, 1000, 2000]$

TABLE II. Hyperparameters for SVM.

Parameter for Decision Tree	Range
criterion	[gini, entropy]
max_depth	$[4, 5, 6, 7, 8, 9, 10, 11, 12, 15, 20, 30, 40, 50, 70, 90, 120, 150]$

TABLE III. Hyperparameters for Decision Tree.

- **Multi-layer Perceptron** (MLP)

  Deep learning algorithm, which is a set of sequential fully connected linear layers of a neural network. Each such layer is represented by a set of weights that relate the output on a particular neuron with the data received from all neurons of the previous layer. The last level uses the Soft-Max layer to obtain the required probabilities in the case of classification.

Multi-layer Perceptron (MLP) is a fully meshed network, which architecture uses 5 linear layers, expanding in the number of output features towards the middle of the network and narrowing towards the output. In addition to line layers, the *GELU* [S3] activation function, batch normalization and dropout layers are used.

The concrete architecture of the model is given below:

```
----------------------------------------------------------------
        Layer (type)               Output Shape         Param #
================================================================
            Linear-1                  [4, 128]           2,176
       BatchNorm1d-2                  [4, 128]             256
              GELU-3                  [4, 128]               0
            Linear-4                  [4, 512]          66,048
       BatchNorm1d-5                  [4, 512]           1,024
              GELU-6                  [4, 512]               0
            Linear-7                  [4, 128]          65,664
       BatchNorm1d-8                  [4, 128]             256
              GELU-9                  [4, 128]               0
          Dropout-10                  [4, 128]               0
           Linear-11                   [4, 64]           8,256
      BatchNorm1d-12                   [4, 64]             128
             GELU-13                   [4, 64]               0
          Dropout-14                   [4, 64]               0
           Linear-15                    [4, 4]             260
================================================================
Total params: 144,068
Trainable params: 144,068
Non-trainable params: 0
----------------------------------------------------------------
```

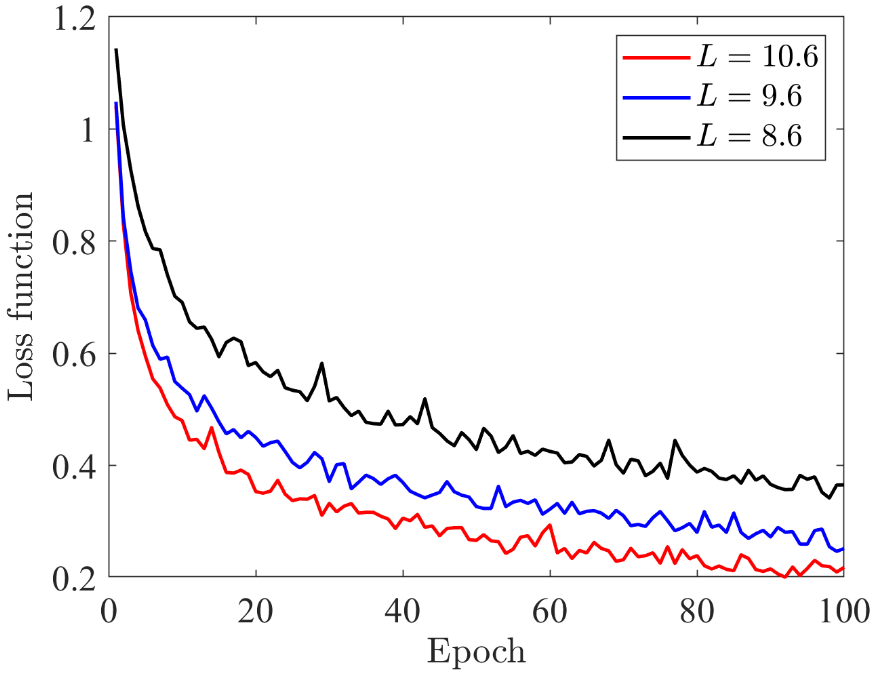

FIG. S6. Loss functions for training data plotted as a function of epoch number for different propagation distances: $L = 8.6$ (black line), $L = 9.6$ (blue line), $L = 10.6$ (red line).

As a machine learning model trains over multiple epochs (100 in our case), the loss function evolves over time. The loss function serves as a measure of how well the model is performing on the given task. A lower loss value indicates better performance and accuracy, as depicted in Fig. S6. The accuracy of the Multilayer Perceptron (MLP) is positively correlated with L, resulting in a smaller plateau of the loss function at high values of epochs.

In the initial epochs, the loss function decreases rapidly as the model learns to identify basic patterns in the data. However, as the model becomes more complex and learns to capture more intricate features, the rate of improvement in the loss function slows down.

- **Convolutional Neural Network** (CNN)

Deep learning algorithm, which is a modification of MLP by adding the so-called convolutional layers. These layers are the same MLP, but have a smaller input size and a smaller amount of output data. Such convolution operations are applied to the input vector with a shift and make sense of spatial trending. Such neural networks do not necessarily show the best accuracy relative to MLP, however, they are more resistant to overlearning/overfitting and, as a rule, are faster.

The convolution operations are applied to the input vector with a shift and make sense of identification of spatial trending. Such neural networks are popular in computer vision tasks, compared to MLPs, they are more resistant to overfitting and, as a rule, faster.

Convolution Neural Network (CNN) — convolutional-mesh network containing 3 one-dimensional convolutional layers combined into one block with batch normalization and activation function *GELU*. After that, there is a global averaging over spatial features (Global Average Pooling) and the passage through the linear layers.

The concrete architecture of the model is given below:

```
----------------------------------------------------------------
        Layer (type)               Output Shape         Param #
================================================================
            Conv1d-1               [4, 32, 16]             128
       BatchNorm1d-2               [4, 32, 16]              64
             Block-3               [4, 32, 16]               0
            Conv1d-4               [4, 64, 16]           6,208
       BatchNorm1d-5               [4, 64, 16]             128
             Block-6               [4, 64, 16]               0
            Conv1d-7              [4, 240, 16]          46,320
       BatchNorm1d-8              [4, 240, 16]             480
             Block-9              [4, 240, 16]               0
AdaptiveAvgPool1d-10               [4, 240, 1]               0
          Flatten-11                  [4, 240]               0
          Dropout-12                  [4, 240]               0
           Linear-13                  [4, 480]         115,680
      BatchNorm1d-14                  [4, 480]             960
             GELU-15                  [4, 480]               0
          Dropout-16                  [4, 480]               0
           Linear-17                  [4, 120]          57,720
      BatchNorm1d-18                  [4, 120]             240
             GELU-19                  [4, 120]               0
          Dropout-20                  [4, 120]               0
           Linear-21                    [4, 4]             484
================================================================
Total params: 228,412
Trainable params: 228,412
Non-trainable params: 0
----------------------------------------------------------------
```

IV. CROSS-VALIDATION CHECK

The use of cross-validation control is studied to determine the suitability of the trained models for data different from the training one. We use the cross-validation control map, which is a graphical tool employed in machine learning to illustrate the cross-validation process.

Here we split a dataset by the parameter N and plot the quality metric in Fig. S7. If the quality metric shown does not drop much, we can conclude that the model has a generalizing ability to other data. Our analysis reveals that generalization occurs for a range of N values, which corresponds to attaching a dimer at each end of the lattice. This feature can be attributed to the fact that this addition of elements does not alter the fundamental topology of the lattice.

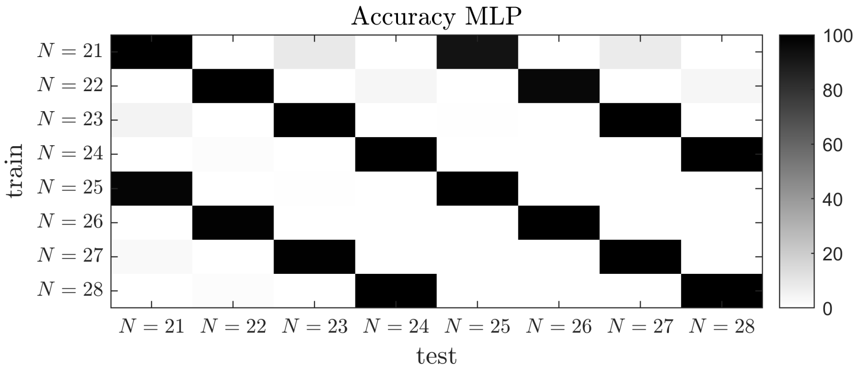

FIG. S7. Cross-validation map.

[S1] See https://scikit-learn.org/stable/modules/neighbors.htmlclassification for implementation details.
[S2] See https://scikit-learn.org/stable/modules/tree.html for implementation details.
[S3] D. Hendrycks and K. Gimpel, “Gaussian error linear units (gelus),” arXiv preprint arXiv:1606.08415 (2016).
